# User-Friendly Genetic Conditional Knockout Strategies by CRISPR/Cas9

**DOI:** 10.1155/2018/9576959

**Published:** 2018-06-14

**Authors:** Liangliang Chen, Ying Ye, Hongxia Dai, Heyao Zhang, Xue Zhang, Qiang Wu, Zhexin Zhu, Rapolas Spalinskas, Wenyan Ren, Wensheng Zhang

**Affiliations:** ^1^Cam-Su Genomic Resource Center, Soochow University, Suzhou 215123, China; ^2^The State Key Laboratory of Quality Research in Chinese Medicine, Macau University of Science and Technology, Avenida Wai Long, Taipa, Macau; ^3^Department of Oncology, St. Jude Children's Research Hospital, 262 Danny Thomas Place, Memphis, TN 38105, USA; ^4^Science for Life Laboratory, Division of Gene Technology, KTH Royal Institute of Technology, 106 91 Stockholm, Sweden

## Abstract

Loss-of-function studies are critically important in gene functional analysis of model organisms and cells. However, conditional gene inactivation in diploid cells is difficult to achieve, as it involves laborious vector construction, multifold electroporation, and complicated genotyping. Here, a strategy is presented for generating biallelic conditional gene and DNA regulatory region knockouts in mouse embryonic stem cells by codelivery of CRISPR-Cas9 and short-homology-arm targeting vectors sequentially or simultaneously. Collectively, a simple and rapid method was presented to knock out any DNA element conditionally. This approach will facilitate the functional studies of essential genes and regulatory regions during development.

## 1. Introduction

Gene function analysis is of crucial importance to understanding normal physiology and disease pathogenesis. One of the best approaches to study the function of a gene is to “knock” it out. While simple constitutive knockouts are useful and informative, in many cases it is desirable to engineer conditional loss-of-function models, particularly for genes essential for cell viability or embryonic development. However, the conventional conditional knockout method is time consuming and laborious due to the complicated targeting vector construction, numerous genetic manipulations, as well as the genotyping with Southern blots or long-range PCR [1, 2].

Due to the simplicity and high efficiency, the CRISPR/Cas9 technique has been extensively used for genetic editing [3–5]. RNA-guided nuclease Cas9 efficiently induces double-strand breaks (DSB) at the targeted locus, which could be repaired by two mechanisms: nonhomologous end joining (NHEJ) and homology-directed repair (HDR). NHEJ produces “indel” mutations (insertions or deletions) at the break site, which disrupt the reading frame of the targeted gene and thus result in a loss of function. HDR acts to repair the DSB and generate precise modifications through homologous recombination in the presence of a donor cassette [5]. Recently, Andersson-Rolf et al. employed the CRISPR/Cas9 technique together with invertible elements to conditionally knock out coding genes [6]. However, the method is limited by a specific requirement of the exons where the invertible element is inserted and thus in theory could only be used for part of the coding genes. Therefore, a simple and universal conditional knockout strategy for targeting any genomic region is desirable and important for dissecting the function of coding genes as well as numerous DNA regulatory elements.

In this study, the easy-made targeting vectors are aided by the CRISPR/Cas9 technique to insert LoxP elements on the flanking regions of the targeted loci sequentially or simultaneously, by which conditional gene knockouts are generated, such as *Eed* (*embryonic ectoderm development*) and *SRCAP* (*Snf2*-*related CREBBP activator protein*) in mouse embryonic stem cells (mESCs). Moreover, a putative regulatory region was conditionally knocked out in one step. Overall, a simple method to conditionally target any DNA element was established, which could also be used for gene editing in human cells.

## 2. Materials and Methods

### 2.1. Generation of Short-Homology-Arm Targeting Vectors

Homology arms ranging from 500 bp to 1 kb were amplified using mouse genomic DNA as a template with Phusion High-Fidelity DNA polymerase (M0530S, NEB) and then purified with AxyPrep PCR Clean-up kit according to the manufacturer's protocol. For the *Eed*-*LNL* targeting construct, the 5′ homology arm was enzymatically digested by KpnI and EcoRI, and the 3′ arm was digested by BamHI and SacII. Then, the two arms were ligated to the *LNL* vector. Similarly, the *Eed*-*FNFL* targeting vector was constructed by inserting the KpnI/EcoRI-digested 5′ homology arm as well as the BamHI/SacII-digested 3′ homology arm into the *FNFL* targeting vector. In addition, a 10 kb *LNL* targeting vector was made by inserting the ApaI/SalI-digested 5′ homology arm and the NotI/SacII-digested 3′ homology arm into the *LNL* vector. A 10 kb *FNFL* targeting vector was generated by inserting the KpnI/EcoRI-digested 5′ homology arm and the BamHI/SacII-digested 3′ homology arm into the *FNFL* vector. The *SRCAP-LNL* targeting vector was made by inserting the KpnI/EcoRI-digested 5′ homology arm and the BamHI/SacII-digested 3′ homology arm into the *LNL* vector. The *SRCAP-FNFL* targeting vector was generated through insertion of the KpnI/EcoRI-digested 5′ homology arm and the BamHI/SacII-digested 3′ homology arm into the *FNFL* vector. The sequences of the primers used to produce targeting vectors are available in Table S2.

### 2.2. Construction of gRNA Expression Vectors

Guide RNA (gRNA) was designed using the CRISPR Design Tool (http://crispr.mit.edu/). gRNA sequences are available in Table S2.

### 2.3. Cell Culture

mESCs were cultured on gelatin-coated Petri dishes with ES medium (DMEM supplemented with 10% fetal bovine serum, 2 mM L-glutamine, 50 mg/mL penicillin, 80 mg/mL streptomycin, 0.1 mM 2-mercaptoethanol (Sigma), and 10^3^ units/mL of leukemia inhibitory factor (LIF; Millipore)) at 37°C and 5% CO_2_. Flip08 (Rosa26^FlpO, Cre-ERT2^) mESCs were used throughout the study.

### 2.4. Cell Electroporation and Colony Selection of the Two-Step Targeting Strategy

1 × 10^6^ mESCs were resuspended in Opti-MEM I medium (Invitrogen, 31985-062). For targeting of LNL cassettes, a total of 9 *μ*g of DNA consisting of Cas9 plasmid, sgRNA expression plasmid, and LNL targeting plasmid in equal parts of 3 *μ*g was mixed with the cells and transferred to a nucleofection cuvette (Sigma, lot number 3110) for electroporation with Lonza Nucleofector™ 2b (electroporation conditions: A23 mES cell). Cells were selected by 250 *μ*g/mL neomycin (G418), 48 hours after electroporation. Individual colonies were handpicked and expanded into 96-well plates for subsequent genotyping, six days after selection. Next, targeting clones carrying the homozygous LNL cassettes were treated with 1 *μ*M 4-hydroxytamoxifen (4-OHT) (Sigma) for five days to induce Cre-LoxP-mediated recombination. After genotyping, cells were electroporated with FNFL targeting plasmids, sgRNA expression plasmids, and Cas9 plasmids. After six days of G418 selection, clones were picked up for PCR genotyping to identify the biallelic insertion of FNFL cassettes. Then, these clones were treated with doxycycline (Dox, 1 *μ*g/mL) for five days to induce the FLP-FRT-mediated recombination. Finally, the cells were treated with 4-OHT to induce Cre-LoxP-mediated recombination. In this way, the genomic regions flanked by two LoxP sites were depleted (Figure S1A).

### 2.5. Cell Electroporation and Colony Selection of the One-Step Targeting Strategy

For the one-step targeting, 10 *μ*g of DNA consisting of Cas9 plasmid, sgRNA expression plasmid for LNL targeting, sgRNA expression plasmids for FNFL targeting, LNL targeting plasmid, and FNFL targeting plasmid all at equal parts of 2 *μ*g was mixed together with mESCs for electroporation. Six days after neomycin (250 *μ*g/mL) selection, individual clones were handpicked and amplified in a 96-well plate to identify the simultaneous insertion of LNL and FNFL cassettes in both alleles. Subsequently, the clones were treated with Dox (1 *μ*g/mL) to deplete neomycin resistance cassette in FNFL flanked by FRT sites. Finally, the clones were treated with 4-OHT (1 *μ*M) to induce the deletion of genomic regions flanked by LoxP sites (Figure S1B).

### 2.6. Genomic DNA Extraction and PCR Genotyping

Cells in a 96-well plate were washed with PBS and lysed with 100 *μ*L of lysis buffer (10 mM Tris-HCl pH 8.0, 0.5 mM EDTA, 0.5% Triton X-100, and 0.5 mg/mL proteinase K) for 2–3 hours at 60°C. Then, genomic DNA was precipitated by the precipitation buffer (3% of 5 M NaCl in absolute ethanol) for 30 min at room temperature (RT), pelleted, and washed twice with 75% ethanol. The resulting DNA pellet was resuspended in sterile ddH_2_O and subsequently used for PCR experiments. PCR amplicons with 3-terminal “A” overhangs were subcloned into the pGEM-T Easy Vector (Promega) for sequencing.

### 2.7. RT-qPCR

Total RNA was isolated from mESCs using TRIzol reagent (Invitrogen). 1 *μ*g of total RNA was subjected to reverse transcription with PrimeScript RT reagent Kit (Takara). Real-time PCR was performed using SYBR Premix Ex Taq™ II (Takara). *GAPDH* was used as an internal control gene. All experiments were conducted in duplicates and repeated three times each.

### 2.8. Immunofluorescence

mESCs were plated in a 24-well plate coated with 0.1% gelatin, washed twice with PBS, and fixed in 4% paraformaldehyde (PFA) solution for 20 min at RT. Then, the cells were treated with 0.1% Triton X-100/PBS for 15 min and blocked by 5% donkey serum (Sigma) for 1 h at RT. Subsequently, the primary antibody H3K27me3 (Abcam) was incubated with cells for 2 hours at RT followed by 1 h incubation at RT with the secondary antibody (Alexa Fluor® 594, Invitrogen). Finally, DAPI (1 : 1000; Sigma) was added followed by incubation of 10 min at RT. The cells were photocaptured with Olympus IX83.

## 3. Results

### 3.1. CRISPR/Cas9-Mediated Conditional Targeting in mESCs

**Figure 1:**
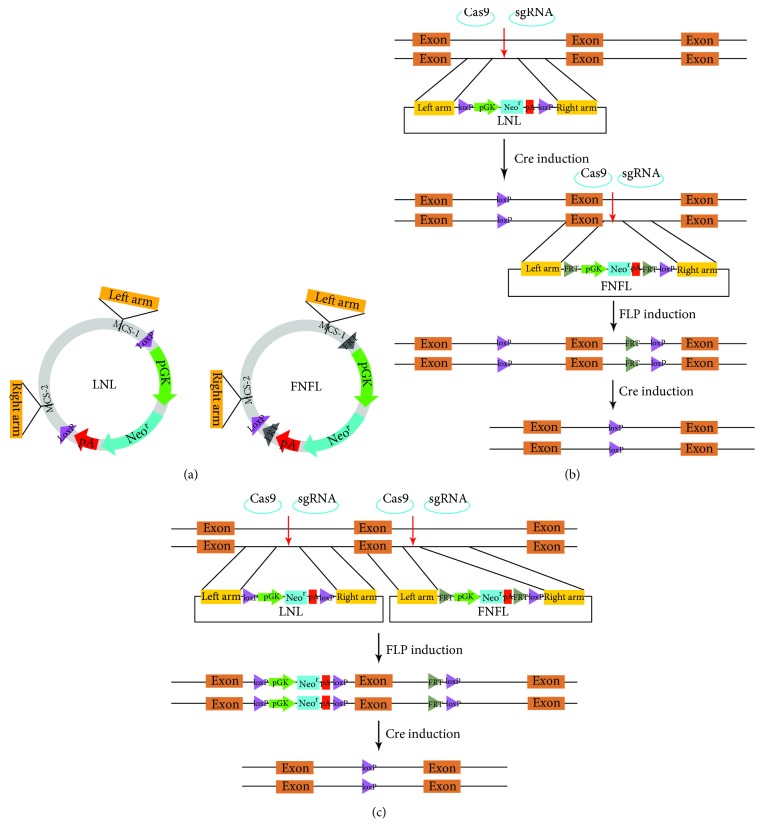
Schematic of CRISPR/Cas9-mediated conditional targeting. (a) Plasmid map of LNL and FNFL vectors. Schematic diagrams of LNL plasmid and FNFL plasmid. LoxP sites are shown by purple arrowheads, and FRT sites are indicated by grey arrowheads. (b) Sequential targeting of LNL and FNFL cassettes in two steps. First, the LNL cassette was inserted to the 5′ site of the region intended to delete. And then the neo^r^ cassette was removed upon 4-OHT treatment, with one LoxP site left. Subsequently, the FNFL cassette was inserted to the 3′ site of the target region. Next, part of the FNFL cassette was depleted by FRT-FLP recombination upon Dox treatment, with one FRT site and one LoxP site left. Finally, the floxed exon was eliminated by Cre-LoxP recombination induced by 4-OHT treatment. LoxP sites and FRT sites are indicated by purple and grey arrowheads, respectively. The CRISPR targeting sites are labelled by red arrows. (c) Simultaneous targeting of LNL and FNFL cassettes in one step. The CRISPR/Cas9 technique was employed to introduce biallelic insertion of LNL and FNFL cassettes to the targeting locus simultaneously. The FNFL cassette was depleted by Dox-inducible FLP-FRT recombination, with one FRT site and one LoxP site being left. Lastly, the LoxP-flanked genomic region was deleted by 4-OHT treatment to induce knockout.

To generate conditional knockouts in mESCs, two strategies were designed, sequentially (Figure 1(b)) and simultaneously (Figure 1(c)) targeting two LoxP-containing cassettes to the flanking sites of the region intended to be deleted. One of the LoxP-containing cassettes, LNL, contained a pGK-regulated neomycin selection gene flanked by LoxP (Figure 1(a)). Another cassette, FNFL, had the pGK-regulated neomycin selection gene flanked by FRT and one LoxP (Figure 1(a)). Both cassettes provided neomycin selection immediately after electroporation. Right after that, the selection genes could be removed.

### 3.2. Generation of Conditional Knockout by Sequential Targeting LNL and FNFL Cassettes

**Figure 2:**
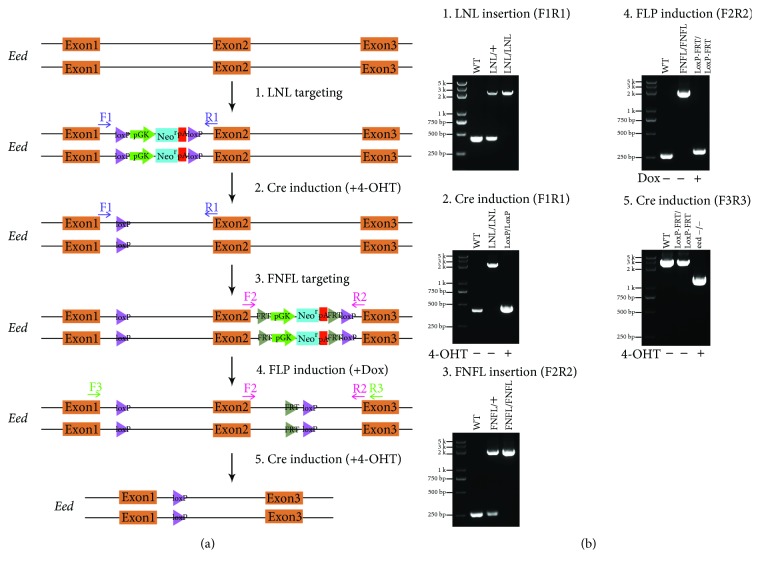
Genotyping of *Eed* conditional knockout generated by a two-step targeting strategy. (a) Schematics of sequential targeting LNL and FNFL cassettes to the *Eed* gene in two steps. (b) The genotyping results of sequential targeting LNL and FNFL cassettes to the *Eed* gene. In clones with biallelic insertion of LNL cassettes, a 2.29 kb DNA fragment was amplified without detecting the 431 bp wild-type amplicon with primers F1 and R1 (b1). When the neo^r^ cassette was depleted upon 4-OHT treatment, we detected a 497 bp PCR amplicon with primers F1 and R1, which was slightly larger than the 431 bp wild-type allele due to the remaining LoxP site (b2). For FNFL homozygous insertion, the PCR amplicon changed from the 263 bp wild-type allele to the 2.16 kb targeting allele using primers F2 and R2 (b3). Next, Dox-inducible FNFL depletion results in the 311 bp DNA fragment, which was slightly larger than the 263 bp wild-type allele because of the remaining LoxP and FRT sites (b4). Finally, when the targeting clones were treated with 4-OHT, a 1.3 kb PCR amplicon was amplified with primers F3 and R3 instead of detecting a 2.38 kb DNA fragment (b5), indicating that the 2nd exon of *Eed* was deleted.

**Figure 3:**
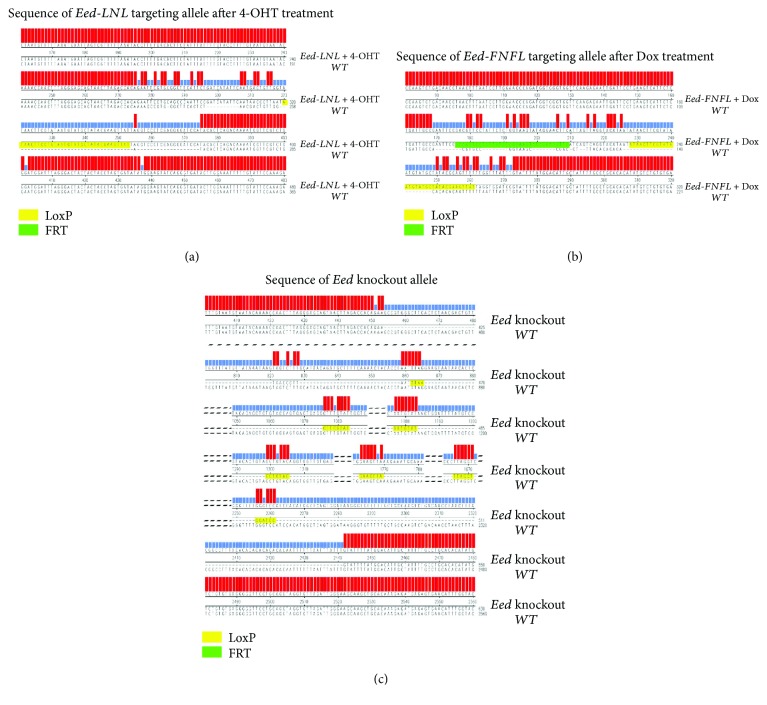
The sequencing result of *Eed* knockout alleles. (a) Sequence of the LNL targeting locus in *Eed* after 4-OHT treatment. The LNL cassette was deleted by 4-OHT-inducible Cre-LoxP recombination, with one LoxP site being left. (b) Sequence of the FNFL targeting locus in *Eed* after Dox treatment. The FNFL cassette was deleted with one LoxP site and one FRT site being left. (c) Sequence of the *Eed* knockout allele generated by 4-OHT-inducible Cre-LoxP recombination. The LoxP site and FRT site are indicated by yellow and green boxes, respectively. The sequence alignment was analyzed by DNASTAR software. The additional mismatched nucleotides are the spacer between the LoxP/FRT site and the restriction endonuclease cut site used for homology arm construction as well as the spacer between LoxP and FRT sites in the FNFL cassette.

To explore the sequential targeting method (Figure 1(b)), the *Eed* gene was selected and its 2nd exon in mESCs was conditionally targeted. Eed is a component of the polycomb repressive complex 2 (PRC2), which represses gene transcription by modulating H3K27me3 [7–9]. The short-arm LNL targeting vector with a 541 bp left arm and 530 bp right arm (Table S1) was produced. The LNL targeting vector was electroporated with sgRNA and Cas9 plasmid in mESCs. After selection by neomycin, ES clones with biallelic insertion of LNL cassette in the 1st intron were identified by PCR with F1 and R1 primers (Figure 2(a)). Four clones out of 88 produced a 2.29 kb DNA fragment after amplification (Figure 2(b). b1, Table 1(a)), indicating the biallelic insertion of LNL cassettes. Then, the LNL biallelic targeting clones were treated with 4-OHT to induce the removal of neomycin resistance cassette, confirmed by the amplification of a 497 bp fragment (Figure 2(b). b2). This fragment was then cloned into the pGEM-T vector for sequencing. As expected, the sequencing results revealed only one LoxP site remaining in the 1st intron of *Eed* (Figure 3(a)).

To insert the FNFL cassette to the 2nd intron of the *Eed* gene, the clones with biallelic LoxP insertion in the 1st intron were electroporated with the FNFL short-arm targeting vector (Figure 2(a), Table S1). Five clones out of 190 produced a 2.16 kb PCR amplicon using primers F2 and R2, indicating the biallelic insertion of FNFL cassettes (Figure 2(b). b3, Table 1(a)). Next, the neomycin cassette in FNFL was removed by FLP/FRT-mediated recombination upon Dox treatment. As expected, only a 311 bp amplicon was retrieved instead of a 2.16 kb fragment (Figure 2(b), b4). The 311 bp fragment was subcloned and sequenced, confirming the depletion of the FNFL cassette with one LoxP site and one FRT site left in the 2nd intron (Figure 3(b)). Finally, the 2nd exon flanked by two LoxP sites was deleted by Cre-LoxP-mediated recombination induced upon 4-OHT treatment. As expected, a 2.38 kb DNA fragment was amplified from the *Eed* conditional knockout cells, while a 1.3 kb fragment was amplified when the 2nd exon was deleted (Figure 2(b), b5, Figure 3(c)).

**Figure 4:**
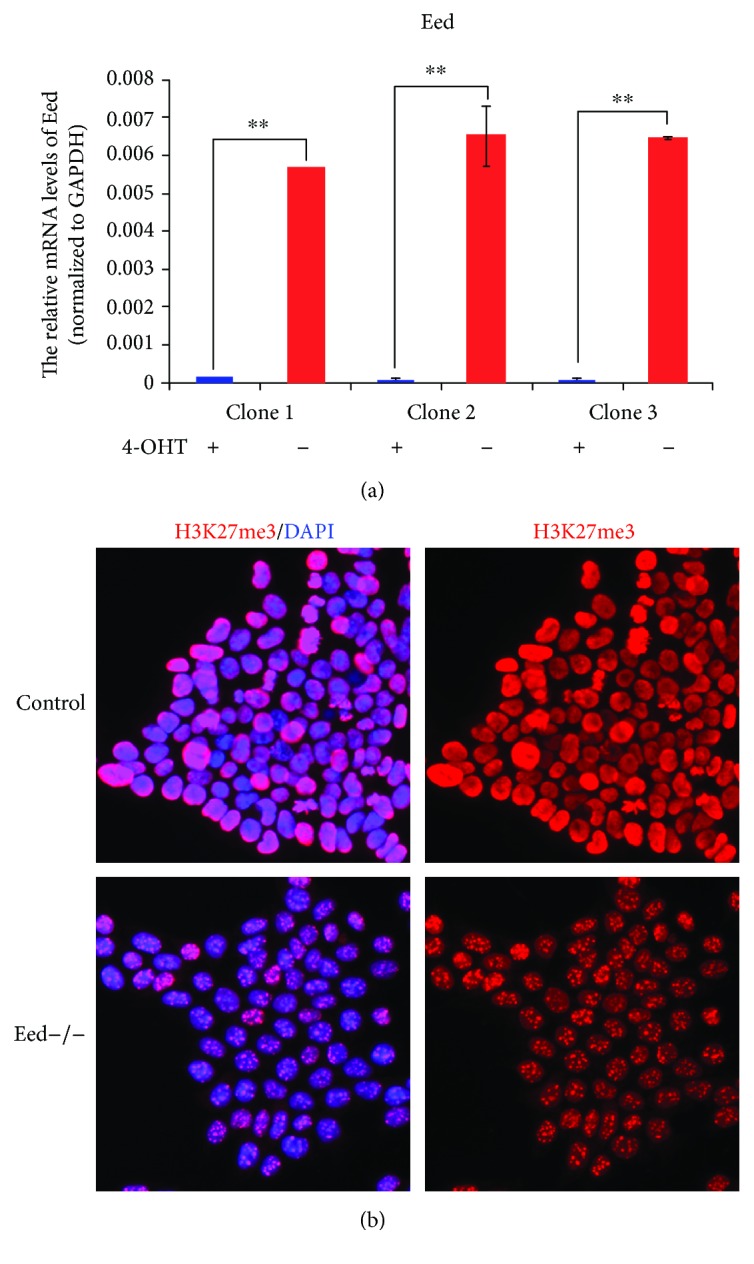
Validation of *Eed* knockout mESCs. (a) RT-qPCR results indicate that transcription was totally abrogated in *Eed* knockout clones generated by 4-OHT treatment. Relative quantification of mRNA levels was performed by the delta Ct (dCt) method and normalized to endogenous GAPDH. Means ± SD are shown. ^∗∗^ *p* < 0.01. (b) Compared to controls, H3K27me3 expression (red, by H3K27) was dramatically decreased in *Eed* knockout mESCs generated by Cre-LoxP recombination upon 4-OHT treatment. Blue indicates DAPI staining.

Furthermore, a RT-qPCR to examine the transcription of *Eed* was performed. As expected, the *Eed* transcription was abolished when the *Eed* fl/fl mESCs were treated with 4-OHT (Figure 4(a)). Additionally, the H3K27me3 modification significantly decreased in *Eed* fl/fl mESCs upon the treatment of 4-OHT (Figure 4(b)), which was consistent with the data reported previously [7, 8].

SRCAP is the subunit of the ATP-dependent chromatin remodeling complex [10, 11]. Using the same strategy, *SRCAP* conditional knockout was generated (Figure S2). Based on the two examples in this study, the LNL biallelic targeting efficiency ranged from 3.2% to 4.5%. The efficiency of FNFL biallelic targeting was from 2.1% to 2.6% (Table 1(a)).

### 3.3. Generation of Conditional Knockout by Simultaneous Targeting LNL and FNFL Cassettes

**Figure 5:**
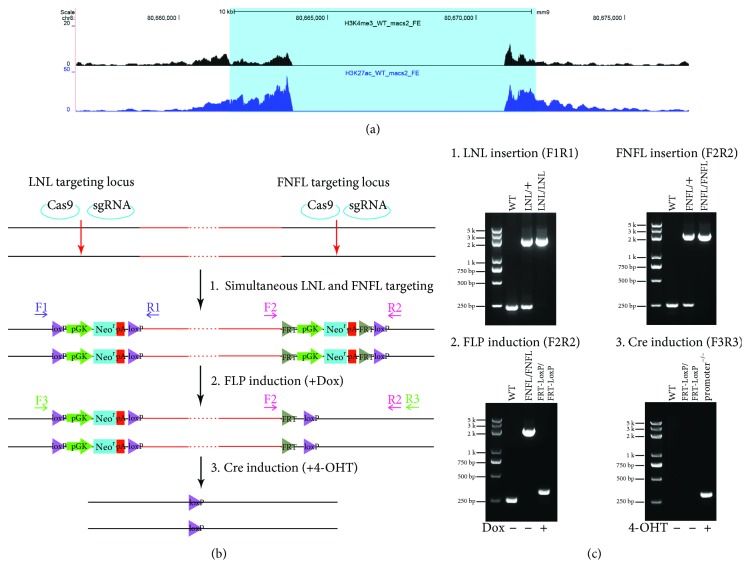
Generation of conditional knockout of one putative regulatory region through a one-step targeting strategy. (a) The 10 kb region is identified as a putative regulatory element based on the H3K27ac and H3K4me3 ChIP-seq data of wild-type mESCs using H3K27ac and H3K4me3 antibodies. H3K4me3 and H3K27ac modifications are shown by black and blue peaks, respectively. (b) Schematics of simultaneous LNL and FNFL targeting to the 10 kb regulatory region in one step. The CRISPR targeting sites for the insertion of LNL and FNFL cassettes are labelled by red arrows. (c) Genotyping results of the 10 kb putative regulatory region with simultaneous targeting of LNL and FNFL cassettes. In clones with biallelic insertion of LNL cassettes, the PCR amplicon change from the 237 bp wild-type allele to the 2.1 kb targeting allele using primers F1 and R1 was detected (c1). Besides, a 2.15 kb amplicon was obtained in FNFL biallelic insertion clones using primers F2 and R2, without detecting the 251 bp wild-type allele (c1). Then, the removal of FNFL cassettes upon Dox addition was identified by the amplication of the 325 bp DNA fragment using primers F2 and R2 (c2). Furthermore, external primers F3 and R3 amplified a 863 bp DNA fragment which could only be amplified when the 10 kb regulatory region was deleted (c3).

Further on, a one-step conditional knockout was tried (Figure 1(b)), for which the LNL and FNFL short-arm targeting vectors were electroporated together to simultaneously target LNL and FNFL cassettes to the flanking sites of a 10 kb putative regulatory element (Figure 5(b), Table S1). The 10 kb region was treated as a putative regulatory element based on its H3K27Ac and H3K4me3 modification pattern [12, 13] from unpublished in-house ChIP-Seq data (Figure 5(a)).

After neomycin selection, the resistant ES colonies were genotyped by PCR to identify the biallelic insertion of LNL and FNFL cassettes. Sixteen clones out of 432 produced a 2.1 kb amplicon with primers F1 and R1, confirming the biallelic insertion of the LNL cassette (Figure 5(c), c1, Table 1(b)). Similarly, 24 clones were genotyped to have biallelic insertion of the FNFL cassette (Figure 5(c), c1, Table 1(b)). So the efficiency of the biallelic insertion of LNL and FNFL cassette was 3.7% and 5.5%, respectively, similar to the biallelic insertion efficiency of the *Eed* and *SRCAP* samples (Table 1). Only one clone out of 432 contained biallelic insertions of both LNL and FNFL cassettes (Table 1(b)). Therefore, the efficiency of simultaneous targeting is still very low and further study is needed to optimize this method in the future.

**Figure 6:**
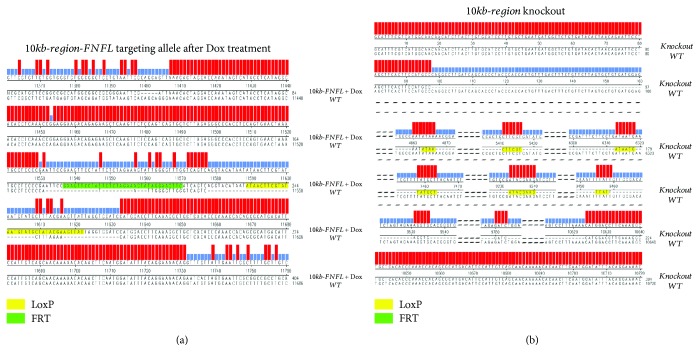
Sequencing results of the conditional knockout allele of the 10 kb putative regulatory region. (a) Sequence of the FNFL targeting locus after Dox treatment. When FLP was induced by Dox, part of the FNFL cassette was deleted with one LoxP site and one FRT site left. The LoxP site and FRT site are indicated in yellow and green, respectively. (b) Sequence of the conditional knockout allele of the 10 kb regulatory region generated by 4-OHT treatment. The sequence alignment was analyzed by DNASTAR software.

Next, the single clone with both cassette insertions was treated with 1 *μ*g/mL of Dox to remove the neomycin resistance cassette in FNFL flanked by two FRT sites. PCR genotyping with F2 and R2 primers produced a 325 bp amplicon resulting from the FLP-FRT-mediated recombination (Figure 5(c), c2). Sequencing results further confirmed the removal of the neomycin resistance cassette with one FRT site and one remaining LoxP site (Figure 6(a)).

The 10 kb fl/fl clone was treated with 4-OHT to delete the 10 kb fragment of the so-called floxed region between two LoxP sites. PCR genotyping was carried out to verify the excision of the floxed region with external primers F3 and R3 (Figure 5(b)). As the DNA fragment in 10 kb fl/fl cells was over 11 kb in length, it was not successfully amplified (Figure 5(c), c3). Instead, a 254 bp DNA fragment was amplified from 10 kb knockout cells (Figure 5(c), c3). Sequencing results further confirmed the deletion of the floxed 10 kb region (Figure 6(b)).

## 4. Discussion

The Cre/LoxP system has been widely applied to generate conditional knockout mutations [14, 15]. However, the conventional knockout technology requires complicated targeting vector construction that normally involves the use of bacterial artificial chromosomes (BAC) and recombineering technology [16, 17]. Moreover, the conventional knockout method is not applicable for human cells due to the low targeting frequency [18].

The development of site-specific nucleases, especially the CRISPR/Cas9 technology, has greatly facilitated the gene editing in various types of cells and organisms owing to its simplicity and robustness, even though the strategy to knock out a gene conditionally is still desirable. Recently, Andersson-Rolf et al.'s research group employed an invertible intronic cassette (FLIP) [6], similar to COIN [19], and generated conditional mutation in a single experimental step. However, due to limitation of the design, this method could only be applied on one half of the whole coding genes theoretically and is not suitable for targeting DNA regulatory elements, namely, promoters and enhancers.

In this study, a targeting strategy mediated by the CRISPR/Cas9 system to generate conditional knockout mutations was developed. Of note, the strategy can be generically applied to target any DNA region conditionally, such as coding genes, noncoding RNA genes, and regulatory elements like promoter and enhancer regions. Compared with the traditional conditional knockout method, our strategy is simple and effective. First, the construction of traditional targeting vectors is time-consuming, which often relies on the genetic manipulation of BAC clones and utilization of recombineering technique. In contrast, the use of short-homology arms (less than 1 kb) in this study makes the assembly of targeting vectors easy and scalable. Second, the traditional method requires multiple steps of genetic manipulation while our simultaneous targeting approach provides a simple and one-step method to conditionally target any DNA element rapidly. Third, the genotyping for the traditional method is complicated as it often involves the use of Southern blot or long-range PCR. Lastly, the efficiency of traditional targeting in human pluripotent stem cells is relatively low and largely dependent on the targeted locus [20, 21]. Alternatively, our strategy may provide a method to generate conditional knockouts in cells of different species.

In this study, conditional knockouts of two coding genes and one putative DNA regulatory region were generated. The simultaneous biallelic targeting efficiency of one LoxP-containing cassette varied between 2.1% and 5.5% in our study. The efficiency for biallelic targeting two LoxP-containing cassettes simultaneously was still very low, which could be improved in future studies by reducing the size of the LoxP-containing cassettes, increasing the sgRNA efficiency, or utilizing a different selection cassette (e.g., puro) for the second targeting vector. Although the targeting efficiency demonstrated in our study is similar to that of the traditional targeting method, our strategy is user-friendly for its simplicity on targeting vector production and genotyping analysis. Furthermore, our method could be optimized further to provide a one-step targeting method with high efficiency in the future.

Altogether, a CRISPR/Cas9-mediated targeting method to generate conditional alleles was established. This design requires only short homology arms with several hundreds of base pairs; thus, it could theoretically be used to target any genomic region. Moreover, it may provide an alternative approach for conditional targeting in human pluripotent cells. The authors believe that this strategy will facilitate the functional studies of essential genes and numerous DNA regulatory elements during the differentiation of pluripotent cells and early embryonic development stages.

## Figures and Tables

**Table 1a:** (a) Sequential targeting of LNL and FNFL cassettes

		Number of clones	Number of heteroinsertion	Number of homoinsertion	Efficiency of heteroinsertion	Efficiency of homoinsertion
*Eed*	LNL insertion	88	17	4	19.3%	4.5%
	FNFL insertion	190	33	5	17.4%	2.6%
*SRCAP*	LNL insertion	94	65	3	69.1%	3.2%
	FNFL insertion	96	70	2	72.9%	2.1%

**Table 1b:** (b) Simultaneous targeting of LNL and FNFL cassettes

		Clones with LNL heteroinsertion	Clones with LNL homoinsertion	Clones with FNFL heteroinsertion	Clones with FNFL homoinsertion	Clones with simultaneous LNL and FNFL homoinsertion
*10 kb promoter*	Number of clones Total clone number :432	194	16	165	24	1
	Efficiency	44.9%	3.7%	38.2%	5.5%	0.2%

## Supplementary Materials

Supplementary 1 Figure S1: detailed procedure of LNL and FNFL sequential and simultaneous targeting. (A) Detailed procedure for sequential targeting LNL and FNFL cassettes in two steps. The first step of LNL cassette targeting is labelled by a red square bracket, and the second step of FNFL cassette targeting is labelled by a blue square bracket. (B) Detailed procedure of simultaneous LNL and FNFL targeting in one step.

Supplementary 2 Figure S2: sequencing results of SRCAP conditional alleles. (A) Sequence of LNL targeting region after 4-OHT treatment. The LNL cassette was deleted by 4-OHT-inducible Cre-LoxP recombination with one LoxP site left. The LoxP site is indicated in a yellow box. (B) Sequence of the FNFL targeting region after Dox treatment. The FNFL cassette was deleted by Dox-induced FLP-FRT recombination, with one LoxP site and one FRT site left. (C) Sequence of the SRCAP knockout allele generated by 4-OHT treatment. LoxP site and FRT sites are indicated in yellow and green boxes, respectively. The sequence alignment was analyzed by DNASTAR software.

Supplementary 3 Table S1: the length of the homology arm of LNL and FNFL targeting vectors.

Supplementary 4 Table S2: sequence of primers and sgRNA used in this paper.
