# Supplementary material for: User-Friendly Genetic Conditional Knockout Strategies by CRISPR/Cas9
Source: Stem Cells Int. 2018 Jun 14;2018:9576959. doi: 10.1155/2018/9576959 (PMC6022269; doi:10.1155/2018/9576959)
Supplement: Supplementary 3 — Table S1: the length of the homology arm of LNL and FNFL targeting vectors. [file 9576959.f3.docx]

**Table S1**

**The length of homology arm of LNL and FNFL targeting vectors**

|  | LNL-left-arm | LNL-right-arm | FNFL-left-arm | FNFL-right-arm |
| --- | --- | --- | --- | --- |
| *Eed* | 541 bp | 530 bp | 743 bp | 755 bp |
| *SRCAP* | 605 bp | 773 bp | 733 bp | 743 bp |
| *10 kb-region* | 872 bp | 812 bp | 668 bp | 759 bp |
