# Supplementary material for: User-Friendly Genetic Conditional Knockout Strategies by CRISPR/Cas9
Source: Stem Cells Int. 2018 Jun 14;2018:9576959. doi: 10.1155/2018/9576959 (PMC6022269; doi:10.1155/2018/9576959)
Supplement: Supplementary 4 — Table S2: sequence of primers and sgRNA used in this paper. [file 9576959.f4.docx]

**Table S2**

| **Primers used for generation of short homology arm targeting plasmids** | |
| --- | --- |
| **Name** | **Primer Sequence (5’ to 3’)** |
|  | ***Eed*** |
| LNL-*Eed*-left-arm F | AATGGTACCGAGGGAGGTGATAGGGCTGA |
| LNL-*Eed*-left-arm R | AATGAATTCTGTGGTCTAAGTTACTGCTCCC |
| LNL-*Eed*-right-arm F | AATGGATCCATACACTCAGACAAAATGGTTCGT |
| LNL-Eed-right-arm R | AATCCGCGGCTAACTGCTCTCCTGCTGGT |
| FNFL-*Eed*-left-arm F | AATGGTACCTATTTACTGCTCACAGGACGAT |
| FNFL-*Eed*-left-arm R | AATGAATTCGGCAATCAGAGAATGACTTC |
| FNFL-Eed-right-arm F | AATGGATCCGTATTTTATGGACATTGCTATTTTGCCTGCAC |
| FNFL-*Eed*-right-arm R | AATCCGCGGCTGGTTTGTATCAACAGTGCTACAC |
|  | ***10 kb putative regulatory region*** |
| LNL-*10 kb*-left arm F | TGGCTTGGAAGAGTCACCTCA |
| LNL-*10 kb*-left-arm R | GGCATGGAAGTGAAGCTAGGAA |
| LNL-*10 kb*-right-armF | TTCTGTTCTTAGTGCTGTGATGGA |
| LNL-*10 kb*-right-arm R | GGTGTCTAGGCCACTTCGTCA |
| FNFL-*10 kb*-left-arm F | GAGGGAGCCGTCATCAGACC |
| FNFL-*10 kb*-left-arm R | GGGGAGGCATTTAGTTCACTGG |
| FNFL-*10 kb*-right-armF | ATGGACCTTCAAAGGCTGCTC |
| FNFL-*10 kb*-right-armR | ACCCAGGATTCAGGACTATCAAA |
|  | ***SRCAP*** |
| LNL-*SRCAP*-left-arm F | gcatgtgctctcctactgaca |
| LNL-*SRCAP*-left-arm R | cagactttggcttggtcattcag |
| LNL-*SRCAP*-right-arm F | gtgactgatatcttttgtgattat |
| LNL-*SRCAP-*right-arm R | atcaatgaaactccaactgtcctta |
| FNFL-*SRCAP*-left-arm F | ccgaactcagaaatctacctac |
| FNFL-*SRCAP*-left-arm R | catgtcagaggcaccaatac |
| FNFL-*SRCAP*-right-armF | gcctggacatgggctct |
| FNFL-*SRCAP-*right-armR | caagcatttacCCGACTGAC |

| **Sequence of gRNAs for LNL and FNFL targeting** | |
| --- | --- |
| **Name** | **sgRNA sequence** |
|  | ***Eed*** |
| *Eed*-LNL targeting gRNA-1 | GGCTTCACTCTAACGACTGTTGG |
| *Eed*-LNL targeting gRNA-2 | AGTCGTTAGAGTGAAGCCCACGG |
| *Eed*-FNFL targeting gRNA-1 | TGTGTAAAGGCCGGCTTACCAGG |
| *Eed*-FNFL targeting gRNA-2 | AGGCCGGCTTACCAGGCACGTGG |
|  | ***10 kb putative regulatory region*** |
| *10 kb*-LNL targeting gRNA-1 | GTAGGGTGCTGATCAAGGCCTGG |
| *10 kb*-LNL targeting gRNA-2 | GGTGGGTAGGGTGCTGATCAAGG |
| *10 kb*-FNFL targeting gRNA-1 | GGACTGACCCAACCCCAATATGG |
| *10 kb*-FNFL targeting gRNA-2 | GACTGACCCAACCCCAATATGGG |
|  | ***SRCAP*** |
| *SRCAP*-LNL targeting gRNA-1 | aggtagtatccctaagtagttgg |
| *SRCAP*-LNL targeting gRNA-2 | atctcttagccaactacttaggg |
| *SRCAP*-FNFL targeting gRNA-1 | gagctcaagccgcagttcgcagg |
| *SRCAP*-FNFL targeting gRNA-2 | agtggggatcctgcgaactgcgg |

| **Genotyping primers** | |
| --- | --- |
| **Name** | **Primer Sequence (5’ to 3’)** |
|  | ***Eed*** |
|  | **LNL targeting in *Eed*** |
| *Eed*-LNL-F1 | TCACTTGTAGGTCACCTGCTTT |
| *Eed*-LNL-R1 | AGCTGCAGGAAATACTGAGAAGT |
|  | **FNFL targeting in *Eed*** |
| *Eed*-FNFL-F2 | TGGCTCAGTGGATAAGGGTGTTT |
| *Eed*-FNFL-R2 | GCAGCTTGCTTCCCAATCTAACA |
|  | ***Eed* knockout allele** |
| *Eed*-knockout-F3 | GAGGGAGGTGATAGGGCTGA |
| *Eed*-knockout-R3 | CTGGTTTGTATCAACAGTGCTACAC |
|  | ***10 kb putative regulatory region*** |
|  | **LNL targeting in *10 kb region*** |
| *10 kb region*-LNL-F1 | GCATTTCGTCATGGCAACAAC |
| *10 kb region*-LNL-R1 | CTCTCTAGCTCCCTGTATCACT |
|  | **FNFL targeting in 10 kb region** |
| *10 kb region*-FNFL-F2 | AAACACTAGGACCAAATAGTCATACCTC |
| *10 kb region*-FNFL-R2 | CTGTTTTCCTGTAAATATCCATTGAAGT |
|  | **10 kb region knockout allele** |
| *10 kb region*-knockout-F3 | GCATTTCGTCATGGCAACAAC |
| *10 kb region*-knockout-R3 | CTGTTTTCCTGTAAATATCCATTGAAGT |

| **qPCR Primers** | |
| --- | --- |
| **Name** | **Primer Sequence (5’ to 3’)** |
| *Eed*-F | GCCAAGAAGCAGAAGTTGAG |
| *Eed*-R | GCATTTGGCGTATTTGTGGG |
| *GAPDH*-F | AGAACATCATCCCTGCATCC |
| *GAPDH*-R | CACATTGGGGGTAGGAACAC |
